# Predicting the status of lymphovascular space invasion using quantitative parameters from synthetic MRI in cervical squamous cell carcinoma without lymphatic metastasis

**DOI:** 10.3389/fonc.2024.1304793

**Published:** 2024-02-06

**Authors:** Limei Guo, Runmei Zhang, Yi Xu, Wenqi Wu, Qian Zheng, Jianting Li, Jun Wang, Jinliang Niu

**Affiliations:** Department of Radiology, The Second Hospital of Shanxi Medical University, Taiyuan, Shanxi, China

**Keywords:** cervical squamous cell carcinoma, lymphovascular space, synthetic magnetic resonance image, relaxation time, proton density

## Abstract

**Purpose:**

To investigate the value of quantitative longitudinal relaxation time (T1), transverse relaxation time (T2), and proton density (PD) maps derived from synthetic magnetic resonance imaging (MRI) for evaluating the status of lymphovascular space invasion (LVSI) in cervical squamous cell carcinoma (CSCC) without lymph node metastasis (LNM).

**Material and methods:**

Patients with suspected cervical cancer who visited our hospital from May 2020 to March 2023 were collected. All patients underwent preoperative MRI, including routine sequences and synthetic MRI. Patients with pathologically confirmed CSCC without lymphatic metastasis were included in this study. The subjects were divided into negative- and positive-LVSI groups based on the status of LVSI. Quantitative parameters of T1, T2, and PD values derived from synthetic MRI were compared between the two groups using independent samples *t*-test. Receiver operating characteristic curves were used to determine the diagnostic efficacy of the parameters.

**Results:**

59 patients were enrolled in this study and were classified as positive (n = 32) and negative LVSI groups (n = 27). T1 and T2 values showed significant differences in differentiating negative-LVSI from positive-LVSI CSCC (1307.39 ± 122.02 vs. 1193.03 ± 107.86, *P*<0.0001; 88.42 ± 7.24 vs. 80.99 ± 5.50, *P*<0.0001, respectively). The area under the curve (AUC) for T1, T2 values and a combination of T1 and T2 values were 0.756, 0.799, 0.834 respectively, and there is no statistically significant difference in the diagnostic efficacy between individual and combined diagnosis of each parameter.

**Conclusions:**

Quantitative parameters derived from synthetic MRI can be used to evaluate the LVSI status in patients with CSCC without LNM.

## Introduction

Cervical cancer is one of the most frequently diagnosed cancers of the female reproductive system, and is a global public health problem (1). Cervical squamous cell carcinoma (CSCC) accounts for approximately 75–80% of all cervical cancers (2). Lymph node metastasis (LNM), as the main metastatic pathway of cervical cancer, is an important independent prognostic factor for cervical cancer (3, 4). Lymphovascular space invasion (LVSI) is an essential step in LNM, reflecting the cancer cells entering the circulatory system (5, 6). Therefore, LVSI is one of the intermediate risk factors in the “Sedlis Criteria” to prompt the clinician to use postoperative radiotherapy after a radical hysterectomy for early-stage cervical cancer (7, 8) and is a crucial factor in determining the best fertility-preserving surgical technique (9). Moreover, LVSI is associated with a higher recurrence rate and decreased 5-year disease-free survival, particularly in patients without LNM (10–12). Thus, early identification of the LVSI status in patients with CSCC without lymphatic metastasis is of great clinical value for optimizing therapeutic strategies to guarantee safety and minimally invasive to minimize the negative effects on the quality of life of patients (13).

At present, the evaluation of LVSI status mainly relies on postoperative pathology. Due to the heterogeneity of tumors, preoperative biopsy cannot accurately evaluate LVSI status (14, 15). Magnetic resonance imaging (MRI) is the preferred modality for the preoperative assessment of cervical cancer. However, conventional MRI allows the visualization of morphological features in cervical cancer and is limited in identifying the LVSI status. Quantitative MRI approaches can provide valuable information for assessing tumor biological behavior. Some studies have preliminarily applied quantitative parameters deriving from such as voxel incoherent motion imaging (IVIM) (16), amide proton transfer weighted imaging (APT) (17) and T2 mapping (18, 19). to predict the LVSI status of cervical cancer. However, the clinical application of these quantitative techniques is prohibited owing to the excessively long scan time.

Synthetic MRI allows simultaneous mapping of multiple relaxometries in a single scan using the multiecho acquisition of saturation recovery using turbo spin-echo readout. It is a novel imaging technique with a significantly shortened overall scan time. As a quantitative and contrast-free technique for determining the biological properties of tissues, this technique is better suited to clinical demands and has been successfully applied to the brain (20, 21), prostate (22), and breast (23) to quantify pathophysiological changes. Thesis researches have shown that the quantitative parameters from synthetic MRI technology can distinguish between benign and malignant tumors and predict the invasiveness. However, the feasibility of synthetic MRI in cervical cancer have not yet been investigated. Therefore, we aimed to investigate the potential of synthetic MRI-derived relaxation maps for predicting LVSI status in CSCC without LNM.

## Materials and methods

### Participants

This prospective study was approved by our institutional Ethics Committee, and written informed consent was obtained from all participants (Number: 2020YXD077). Patients with suspected cervical cancer who underwent pelvic MRI between May, 2020 and March, 2023 were enrolled in this study. Inclusion criteria: patients with a high suspicion of cervical cancer for the first time with strong evidence from clinical examination, ultrasound, MRI or endoscopy. The exclusion criteria were as follows: 1) patients who have not undergone radical hysterectomy; 2) pathologically confirmed LNM or non-CSCC; 3) no visible lesions or lesions with a maximum diameter of < 5 mm; and 4) poor image quality. The FIGO stage, the maximum diameter of the tumor, HPV genotypes, LVSI status, differentiation of tumor, and depth of invasion were collected.

### MRI acquisition

All patients underwent 3.0 T magnetic resonance examinations (Signa Pioneer; GE Healthcare, Milwaukee, WI, USA) equipped with an abdominal coil. Conventional contrast-weighted images were acquired using axial T1-weighted imaging, axial/sagittal T2-weighted imaging, and axial/coronal fat-suppressed T2WI. Sagittal synthetic MRI (Magnetic Compilation, MAGiC) was performed, and the acquisition parameters were as follows: repetition time, 4000 ms; echo time, 18.4/92 ms; inversion time, 210, 610, 1810, and 3810 (ms); flip angle, 90 and 110 (degree); field of view, 260×260 mm; section thickness/gap, 5 mm/1 mm; matrix, 320×192; and acquisition time, 3 min 28s.

### MRI analysis

**Figure 1:**
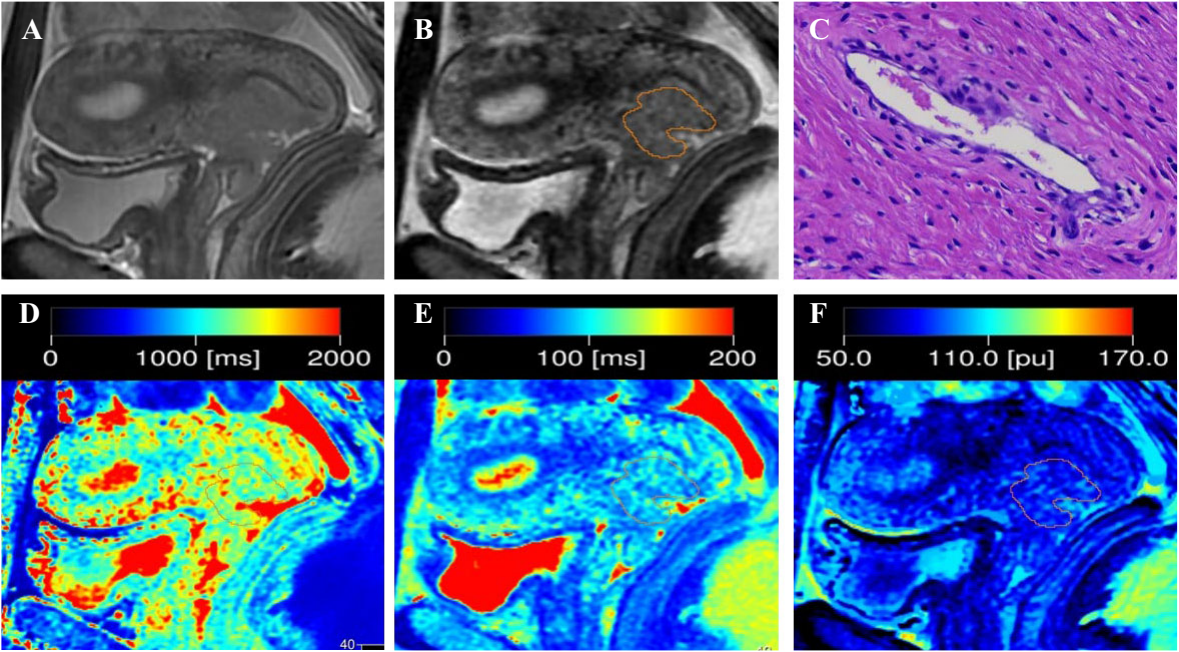
Representative images from a 33-year-old female patient with negative-LVSI cervical squamous cell carcinoma. **(A)** sagittal T2-weighted imaging (T2WI); **(B)** Regions of interest were manually drawn along the border of the tumor on the synthetic T2-weighted images derived from the synthetic MRI sequences; **(C)** hematoxylin–eosin (HE) staining of the tumor specimen (×100) showed no tumor cells were found in the space lined by endothelial cells; **(D)** T1 map, **(E)** T2 map, and **(F)** PD map. T1 value:1356ms, T2 value: 95ms, PD value: 82pu.

**Figure 2:**
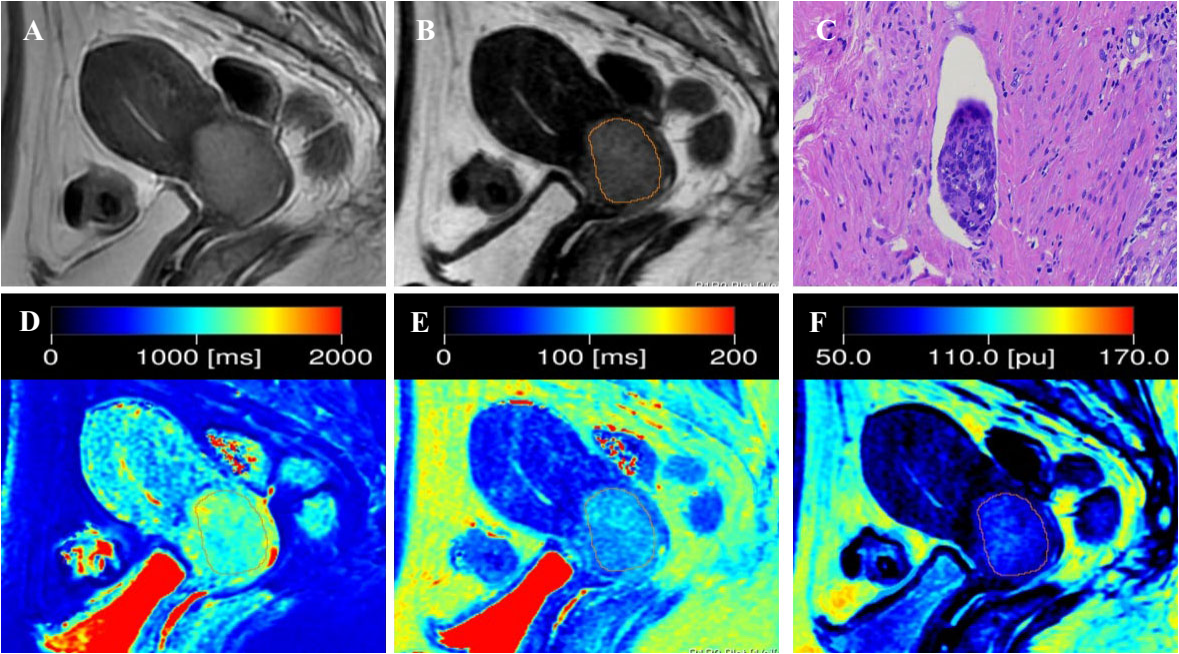
Representative images from a 52-year-old female patient with positive-LVSI cervical squamous cell carcinoma. **(A)** sagittal T2-weighted imaging (T2WI); **(B)** the synthetic T2-weighted image; **(C)** hematoxylin–eosin (HE) staining of the tumor specimen (×100) showed a cluster of tumor cells were found in the space lined by endothelial cells; **(D)** T1 map, **(E)** T2 map, and **(F)** PD map. T1 value:1148ms, T2 value: 82ms, PD value: 79pu.

Quantitative T1, T2, and PD maps were automatically generated with the post-processing software of MAGiC. For synthetic MRI-derived parameters on the synthetic T2-weighted image, the slice with the largest tumor diameter was selected, and the tumor border was delineated (**Figures 1**, **2**). The necrotic, obvious cystic regions, and vessels were excluded by referring to conventional sagittal T2WI.Two radiologists (with 4 and 7 years of experience in MRI), who were blinded to the patients’ clinical information, analyzed the images separately, and the average value was used as the final result.

### Statistical analysis

Intraclass correlation coefficients (ICCs) were calculated to determine the interobserver consistency in T1, T2, and PD measurements from two observers and classified as excellent (>0.80), good (0.60–0.79), fair (0.40–0.59), and poor (<0.40). Continuous data with non-normal distribution were expressed as median (first quartile, third quartile), and data with normal distribution were expressed as mean ± standard deviation. Depending on the data distribution, independent sample *t*-test and the Mann–Whitney *U* test were performed to determine the differences in T1, T2, and PD values among the different LVSI statuses. Receiver operating characteristic (ROC) curve analysis was performed to analyze the diagnostic efficacy. Using MedCalc software for Delong test, compare the AUC values of different parameters in predicting LVSI states. *P*< 0.05 was considered statistically significant. The Statistical Package for the Social Sciences 22.0 (IBM, Armonk, NY, USA) was used for statistical analysis.

### Histopathological analysis

According to the preoperative 2018 FIGO staging of cervical cancer, all patients underwent radical hysterectomy ± bilateral salpingo-oophorectomy + bilateral pelvic lymph node dissection ± para-aortic lymph node dissection. HE staining was performed, along with immunohistochemical staining of CD34 (for evaluating vascular endothelium) and D240 (for evaluating lymphatic vessels). LVSI positivity is defined as the presence of adherent tumor cells in the space lined by endothelial cells on the outer edge of tumor tissue (24, 25). Collect the histopathological features of the patient, including the degree of tumor differentiation, maximum diameter of the tumor, depth of stromal invasion, etc.

HPV DNA tests were conducted on tumor samples using polymerase chain reaction, followed by reverse dot blotting. The types of HPV subtypes tested include 6 low-risk (6, 11, 42, 43, 44, and 81) and 15 high-risk HPV (16, 18, 31, 33, 35, 39, 45, 51, 52, 53, 56, 58, 59, 66, and 68) genotypes (26).

## Results

### Patients demographics

**Figure 3:**
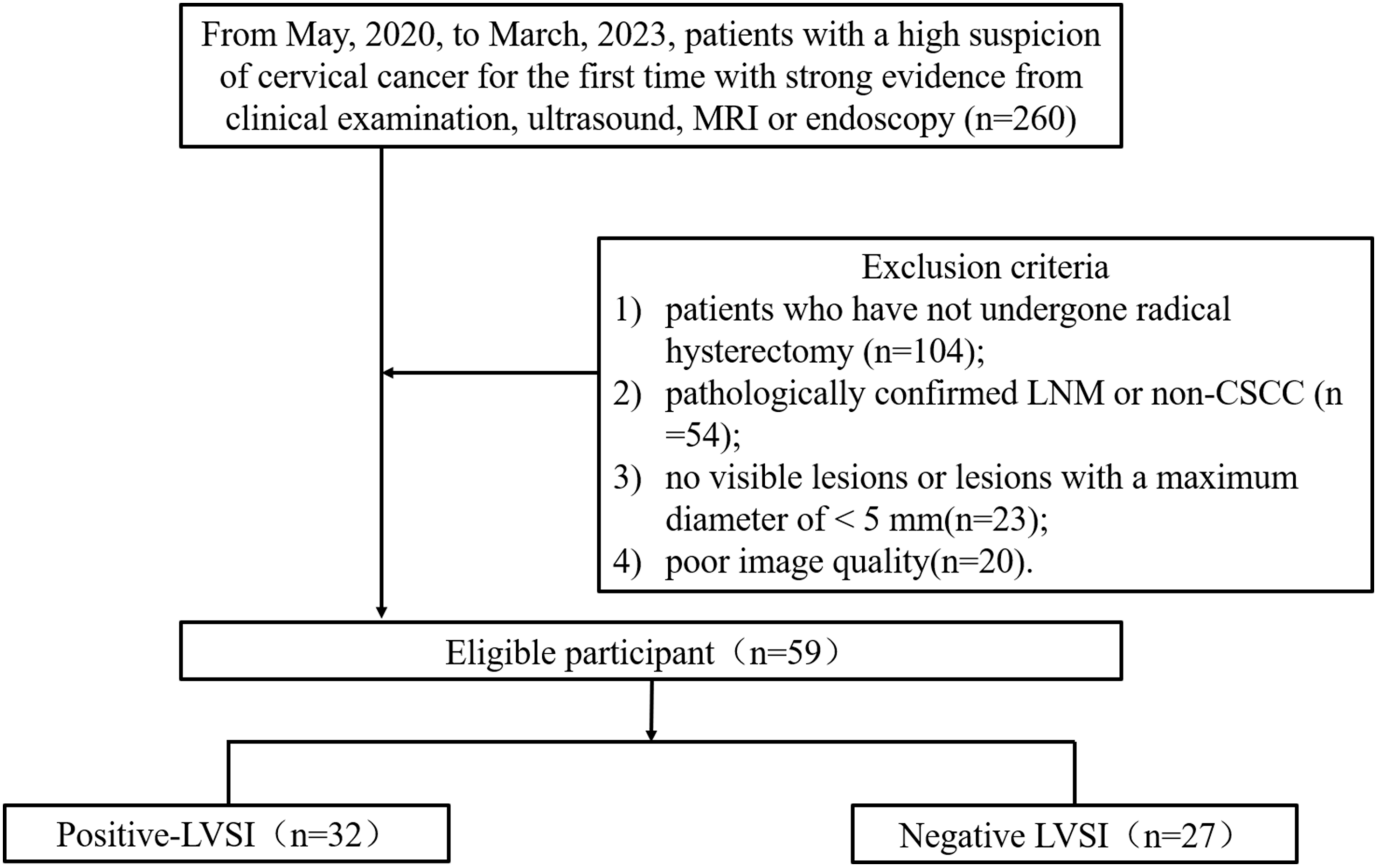
Patient selection.

Fifty-nine patients with CSCC were enrolled in the analysis, namely, 32 with positive LVSI and 27 with negative LVSI (**Figure 3**). The clinicopathological characteristics of the study population are shown in **Table 1**. The depth of stromal invasion differed significantly between the positive and negative LVSI groups (*P* = 0.014). No significant differences in age, FIGO stage, grade of differentiation, maximum tumor diameter, or HPV genotypes were found between the positive and negative LVSI groups (all *P*> 0.05).

**Table 1:** Clinical characteristics.

	Negative-LVSI (n = 27)	Positive-LVSI(n=32)	*P*
**Age(years)**	49.8 ± 14.4	52.7 ± 9.7	0.378
FIGO stage			
IB	22	24	0.550
IIA	5	8	
Differentiation			
Poor	10	13	0.778
Well/moderate	17	19	
Maximum diameter			
<2 cm	3	6	0.705
≥2 cm and <4 cm	18	19	
≥4 cm	6	7	
Depth of stromal invasion			
Superficial 1/3	9	2	0.014
Middle 1/3	8	8	
Deep 1/3	10	22	
High-risk HPV			
Positive	26	29	0.731
Negative	1	3	

LVSI, lymphovascular space invasion.

### Interobserver agreement for quantitative T1, T2, and PD measurements

The T1, T2, and PD values of patients with CSCC showed convincing interobserver agreement. The ICC for T1, T2, and PD measurements were0.876 (95% confidence interval [CI], 0.800–0.924), 0.866(95% CI, 0.784–0.918), and 0.886(95% CI, 0.815–0.930), respectively.

### Quantitative parameters for assessment of LVSI

**Figure 4:**
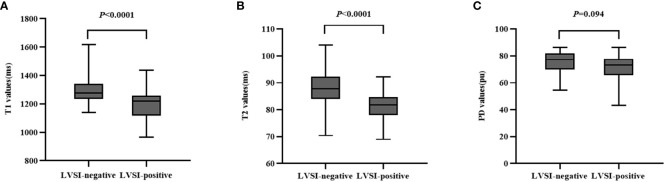
Box-and-whisker plots show the quantitative parameters derived from synthetic MRI based on different lymphovascular space invasion status of cervical squamous cell carcinoma patients without lymphatic metastasis **(A–C)**.

In positive-LVSI CSCC groups, significantly lower T1 and T2 values were observed than those in the negative groups (T1: 1193.03 ± 107.86 vs. 1307.39 ± 122.02, *P*<0.0001 and T2: 80.99 ± 5.50 vs. 88.42 ± 7.24, *P*<0.0001). There was no significant difference in PD values between the LVSI (-) and LVSI (+) groups (*P*=0.094). The T1, T2, and PD values for different LVSI statuses are shown in **Table 2** and **Figure 4**.

**Table 2:** Assessment of quantitative parameters derived from synthetic magnetic resonance imaging in different lymphovascular space invasion status of patients with cervical squamous cell carcinoma patients without lymphatic metastasis.

Groups	T1 (ms)	T2 (ms)	Proton density (pu)
Positive (n=32)	1193.03 ± 107.86	80.99 ± 5.50	71.09 ± 9.94
Negative (n=27)	1307.39 ± 122.02	88.42 ± 7.24	75.24 ± 8.56
*P* value	<0.0001	<0.0001	0.094

### Diagnostic performance of each quantitative parameter in distinguishing LVSI status of CSCC patients without LNM

**Figure 5:**
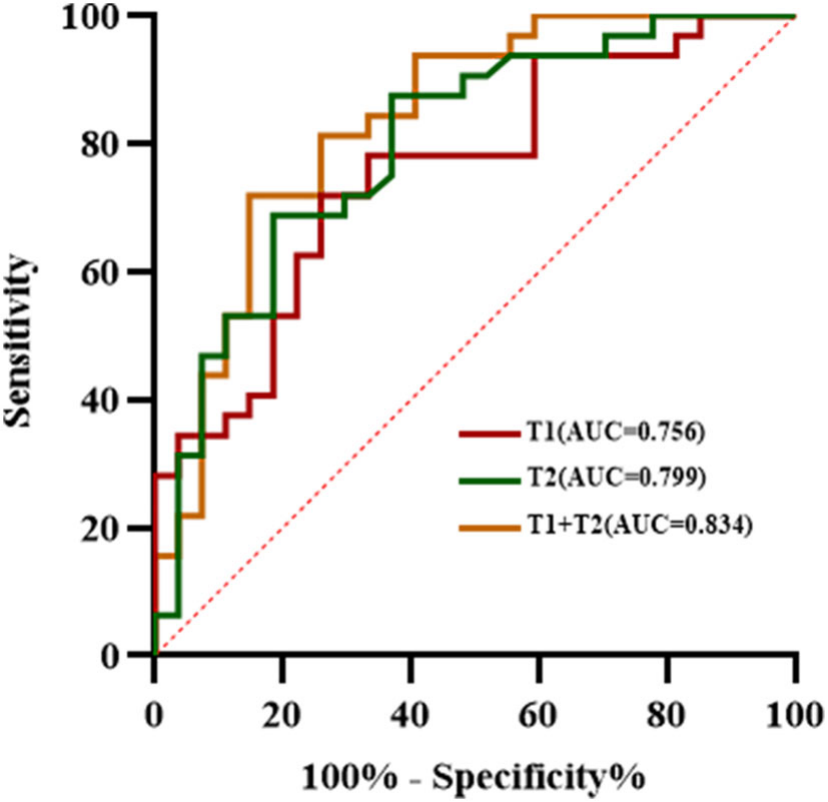
The receiver operating characteristic analysis results of T1, T2 values and combined T1 and T2 in the matter of lymphovascular space invasion status in cervical squamous cell carcinoma without lymphatic metastasis.

The ROC analysis results of each quantitative parameter for distinguishing the LVSI status are shown in **Table 3** and **Figure 5**. In terms of discriminating positive-LVSI from negative-LVSI CSCC, a T1 value of 1240.425ms and T2 value of 85.3505ms were the most accurate cut-off levels. It was found that the combined diagnosis of T1 and T2 values had a better diagnostic efficacy of 0.834. Using Delong test to compare the AUC values of different parameters predicting LVSI status, it was found that there was no statistically significant difference between T1 and T2 combined diagnosis and T1 and T2 values analyzed separately (*P*=0.123, *P*=0.281).

**Table 3:** Diagnostic performance of T1 and T2 values in assessing the status of lymphovascular space invasion in cervical squamous cell carcinoma without lymphatic metastasis.

Parameters	AUC	Sensitivity	Specificity	Cutoff (ms)
T1 value	0.756	0.719	0.741	1240.425
T2 value	0.799	0.875	0.630	85.3505
T1+T2 value	0.834	0.719	0.852	–

AUC, area under the curve.

## Discussion

In this study, we explored the feasibility of a synthetic MRI technique for predicting the LVSI status in CSCC without LNM. T1 and T2 values significantly differed between positive and negative LVSI in CSCC without LNM. Our results demonstrate that the quantitative relaxation metrics derived from synthetic MRI could offer vital information to aid clinicians in creating individualized treatment plans for CSCC.

T2WI is an important routine MR sequence for clinical evaluation of cervical cancer, and is often used for qualitative research. T2 mapping is the technique that enables direct quantification of potential pathophysiological processes in biological tissues and T2 relaxation time has been recognized reflecting the mobile water content in different tissues (27). T2 values have been used to identify malignant lymph nodes in rectal cancer (28), distinguish the levels of tumor differentiation of renal cell carcinoma (29) and differentiate between prostate carcinoma and chronic prostatitis (30). In terms of cervical cancer, previous studies have explored the role of T2 mapping in predicting the status of LVSI (18, 19). Li demonstrated the usefulness of T2 values derived from a radial turbo-spin-echo T2 mapping sequence to identify LVSI status in CSCC (18). In our study, we found a significantly lower T2 value in CSCC with LVSI, which is consistent with Li’s previous works. However, the AUC value of in our study is lower than Li’s results, this difference may be explained by the different patient cohorts. Consistent with the results of previous studies, our results confirmed that the T2 value was an effective tool for identifying the LVSI status in CSCC.

T1 mapping has been used as an essential clinical tool to assess diffuse myocardial fibrosis and predict a poor prognosis (31). Recently, it has shown potential in differentiating grades of renal cell carcinoma (32), identifying extramural venous invasion status in rectal cancer (33), and predicting the recurrence of hepatocellular carcinoma (34). Wang et al. (35) demonstrated that extracellular volume measurements based on T1 mapping performed well in discriminating between patients with cervical cancer with and without LVSI. In this study, we investigated the usefulness of the T1 value for predicting the LVSI status in CSCC. Our results demonstrated that the T1 values significantly differed between LVSI-positive and LVSI-negative CSCC. Tissue T1 relaxation time is associated with various biological factors, such as macromolecule concentration and water-binding status (36). LVSI-positive tumors are more prone to aggravation of hypoxia, cellular necrosis and the accumulation of macromolecular substances (16). These factors may account for the shorter T1 relaxation times in positive-LVSI tumors than those in negative-LVSI tumors. Our study found that PD value was not significantly different between the different LVSI status subgroups. A study with a larger sample size is warranted to explore the role of the PD value in predicting the prognostic factors of CSCC.

Synthetic MRI can improve the efficiency compared to those of other conventional T1 and T2 mapping. In the present study, both T1 and T2 values were useful quantitative parameters for predicting the LVSI status in CSCC, although there is no significant improvement of the diagnostic performance in predicting LVSI in CSCC using the combination of T1 and T2 values.

This study had several limitations. First, the sample size was small. Therefore, a multicenter study with a large cohort is required. Second, in this study, the region of interest was only delineated at the maximum level of the tumor, which might have introduced some bias owing to the heterogeneity of the tumors. Finally, T1, T2, and PD maps are complex variables that reflect tissue properties, and the relationship between these parameters and pathological features needs to be further explored in the future.

In conclusion, this study showed that synthetic MRI could help discriminate the invasion status of the lymphovascular space in patients with CSCC without lymphatic metastasis, and can noninvasively predict the LVSI status before surgery to help clinicians in treatment decision-making and estimate prognosis.

## Data availability statement

The raw data supporting the conclusions of this article will be made available by the authors, without undue reservation.

## Ethics statement

The studies involving humans were approved by Ethics Review Committee of the Second Hospital of Shanxi Medical University. The studies were conducted in accordance with the local legislation and institutional requirements. The participants provided their written informed consent to participate in this study. Written informed consent was obtained from the individual(s) for the publication of any potentially identifiable images or data included in this article.

## Author contributions

LG: Conceptualization, Investigation, Writing – original draft, Writing – review & editing. RZ: Data curation, Methodology, Writing – original draft. YX: Data curation, Methodology, Writing – original draft. WW: Formal analysis, Methodology, Writing – original draft. QZ: Formal analysis, Methodology, Writing – original draft. JL: Formal analysis, Methodology, Writing – original draft. JW: Resources, Supervision, Writing – review & editing. JN: Conceptualization, Project administration, Supervision, Writing – review & editing.
